# The transition to retirement and subsequent physical health among middle-aged and older adults in China: A life course perspective

**DOI:** 10.1371/journal.pone.0347550

**Published:** 2026-04-24

**Authors:** Guodong Zhu, Dianxi Wang, Shijun Chen

**Affiliations:** 1 China Wushu School, Beijing Sport University, Beijing, People’s Republic of China; 2 School of Marxism, Beijing, Beijing Sport University, Beijing, People’s Republic of China; 3 School of Humanities, Beijing Sport University, Beijing, People’s Republic of China; Folkhälsan Research Center, FINLAND

## Abstract

The transition from work to retirement is a critical shift in an individual’s later life. Utilizing retrospective data from the 2014 China Health and Retirement Longitudinal Study (CHARLS), this study employed sequence analysis to identify typical retirement transition patterns, followed by multiple regression analysis to examine their association with physical health. Five groups of retirement trajectories are identified: “Not Retired,” “Early Retirement,” “Late Retirement from Agricultural Employment,” “Statutory Retirement,” “Late Retirement from Self-employment”. The “Statutory Retirement” and “Early Retirement” groups typically complete their retirement transition earlier, often retiring at the statutory age with substantial pension support. In contrast, the “Not Retired,” “Late Retirement from Agricultural Employment,” and “Late Retirement from Self-employment” groups exit the labor force later in life. These individuals are primarily engaged in agricultural production or self-employment and generally lack robust pension insurance. Regression analysis found a significant association between retirement transition patterns and physical health. The “Statutory Retirement” group demonstrates lower Instrumental Activity of Daily Living (IADL) (*B* = −0.393, *P* < 0.001), and NAGI (*B* = −0.791, *P* < 0.001) scores. The “Late Retirement from Self-employment” group also showed lower IADL (*B* = −0.356, *P* < 0.01) and NAGI (*B* = −0.555, *P* < 0.001) scores. The “Early Retirement” group exhibits a lower NAGI score (*B* = −0.345, *P* < 0.001). In contrast, the “Late Retirement from Agricultural Employment” group was associated with higher scores in Activities of Daily Living (ADL) (*B* = 0.212, *P* < 0.05) and IADL (*B* = 0.407, *P* < 0.01). Contact with children moderated the relationship between retirement transition and physical health. Increased intergenerational contact had a stronger positive correlation with health for individuals in the “Late Retirement from Agricultural Employment” group but showed no significant health benefit for those in the “Statutory Retirement” group.

## 1. Introduction

During the transition into old age, events such as children leaving the parental home, retirement from paid work, the loss of a spouse or partner (i.e., bereavement), and changes in residential location are considered important life events in later life [1–2]. These events entail transitions in associated social roles or statuses, and variations in their timing and duration may contribute to the formation of distinct life trajectories for individuals as they transition into old age.

Retirement transition constitutes a pivotal event and an obvious change of status in later life [3], where individuals navigate substantial shifts in their social position, personal identity, and associated responsibilities [1]. Recent studies have shown that, with changes in socioeconomic conditions, the transition from work to retirement in Western societies has undergone profound transformations, characterized by increasing diversity and de-standardization [4]. In the Chinese context, the gradual aging of the baby-boom generation post-2020 is leading a growing workforce into retirement. Studies on older adults in China have also found that the retirement process exhibits complex and varied patterns [5–6]. Given the importance of retirement within the broader transition to old age, this study focuses specifically on retirement as a life event and the process of transitioning to retirement.

Given rapid population aging and longer retirement periods, promoting health and well-being in later life has become an increasingly critical objective. Extensive research has investigated retirement as a life event with significant health implications for older adults [7–9]. However, most studies treat retirement as a discrete event affecting subsequent health outcomes, rather than examining it as an extended process within the life course. In reality, retirement is not an isolated occurrence, but a transitional process involving multiple concurrent states. Meantime, health changes during retirement are also not abrupt phenomena, but rather the cumulative expression of lifelong advantages or disadvantages shaped by socioeconomic status and work experiences. This process unfolds within China’s unique social context, characterized by its urban-rural dual system and familial obligations such as intergenerational caregiving. By shifting the focus from retirement as an immediate event to an integrated life-course process, this study adopts a critical holds a critical perspective. It utilizes retrospective life history data to conceptualize the work-to-retirement shift as a multi-state life course transition, examining the retirement patterns of Chinese individuals born between 1920 and 1960 and their association with later-life physical health.

## 2. Theoretical foundations

### 2.1. Transition to retirement: As a process

The conceptualization of retirement in the existing literature has evolved significantly. Early scholarship predominantly defined retirement either as a discrete event or a transition between states [10], or as a one-time, complete withdrawal from the labor market driven by personal choice or preference [11–12]. Within these frameworks, retirement was understood as a definitive entry into a status devoid of paid employment and complete detachment from labor market participation. Therefore, many scholars viewed retirement as a pivotal life-course transition that marks the shift from a prolonged phase of continuous work to a period characterized by leisure activities [13], signifying the onset of old age and a period of inactivity [14].

Retirement, however, is not necessarily a sudden and complete withdrawal from paid labor. It can instead be a gradual process of labor market exit, which may involve progressively reducing working hours, transitioning to less demanding jobs, or engaging in part-time work while drawing a pension [15]. This process may also include scenarios where individuals continue to perform a limited amount of paid work weekly after reaching the statutory retirement age. Therefore, retirement may manifest in multiple forms. For example, unemployed individuals may be compelled to exit the labor market due to their unemployment status, those retiring from stable full-time careers may experience a relatively brief transition, while others who opt for continued part-time work may undergo a more extended and gradual retirement phase. Thus, the conceptualization of retirement as a simple and abrupt state transition appears reductive. As proposed by Atchley [16], retirement adjustment may be a dynamic, multi-stage development process. Empirical evidence strongly supports this view. Research consistently indicates that retirement is rarely a straightforward, one-time transition from employment to pension receipt; rather, it unfolds over several years [17–19]. This prolonged process involves a series of changes in work-related behaviors and labor market engagement [20–21], encompassing varied trajectories both within and outside the formal labor market [22]. Such extended transitions can provide opportunities for individuals to cultivate new identities, roles, and lifestyles [23–24]. Some scholars further argue that retirement can be broadly classified into narrow and broad definitions, namely “narrow retirement based on formal labor and broad retirement based on informal labor, and diverse retirement interwoven both” [5^:^80]. Narrow retirement aligns with a “standard” exit based on formal labor arrangements, whereas broad retirement corresponds to a “diverse” range of pathways that include engagement in informal labor.

Moreover, the forms and pathways of retirement are often complex, encompassing various patterns such as mandatory, partial, and phased or gradual retirement [25]. It is important to note that some retirement paths do not entail a complete withdrawal from the labor market [26–27]. Therefore, from a holistic perspective, retirement should not be understood as a concentrated, one-time change in status, but rather as a potentially dispersed and long-term process, which can even be regarded as a new life stage or the onset of a “third age” in adulthood [3,28]. Combining the above discussions, this study adopts the view of retirement as a dynamic process and conceptualizes retirement as a series of dynamic changes in roles or states which older adults move from full or stable employment toward permanent or ultimate labor market exit. Thus, retirement is not a simple, singular shift from employment to labor market exit, but a more complex dynamic trajectory characterized by multiple status changes. Therefore, this study operationalizes the transition to retirement as a dynamic process of gradual labor market withdrawal, involving variations in labor force participation, employment status, and social roles across different ages.

### 2.2. Changes in the transition to retirement

Since the late 1990s, the impending retirement of the post-World War II baby boom generation, combined with declining fertility rates and rising life expectancy, has placed significant pressure on societies to support aging populations [29]. Therefore, countries facing population aging have, since the 1990s, implemented a series of policy measures aimed at increasing labor force participation among older adults. These measures include closing pathways to early retirement, eliminating mandatory retirement ages, raising the eligibility age for state pension, permitting part-time work while receiving pension benefits, and enacting legislation against age discrimination in the labor market [30–31]. Within this policy context, the trend toward early labor market exit among older adults in many developed countries has reversed, leading to increased career continuity. For example, in 2010, more than 80% of men aged 55–59 in Denmark, Sweden, and Switzerland remained employed, while employment rates for this group also reached 70% in Austria, Belgium, France, and Italy. For women aged 55–59, employment rates exceeded 60% in the United States, Switzerland, Sweden, Denmark, Germany, and the United Kingdom, and were around 50% in Spain, Italy, Belgium, and Austria [32].

Rising labor force participation among older adults is reshaping both the timing and pathways of retirement. Increasingly, individuals diverge from a uniform exit at a statutory age; some postpone retirement due to policy mandates, economic necessity, or personal choice, while others exit earlier due to health issues, involuntary job loss, or despite being eligible for retirement but do not wish to retire. Empirical studies also confirm that a complete and permanent exit from work is no longer the sole, or even the dominant, pattern [33]. Especially as individuals approach retirement, they often navigate a series of complex, multi-year transitions. These may encompass delayed retirement, partial retirement (reduced hours or roles), and “bridging jobs” that span the period between full-time work and full labor force withdrawal [34–35]. The prevalence of such pathways is substantial. For example, Reimers and Honig [36] found that 37% of men opted for partial retirement, and Ruhm’s [37] estimated that about half of individuals experience it at some point in their lives. Using data from the U.S. Health and Retirement Study, Maestas [25] reported that from 1992 to 2002, nearly 50% of older Americans followed non-traditional retirement pathways such as gradual or partial retirement. Similarly, based on Germany survey data, Fasang [4] found that among cohorts born 1932–1949 (observed 1991–2006), only 18% retired directly from full-time work, while 34% experienced unemployment before retirement, and 48% via other diverse pathways. In sum, the emergence of these varied forms and trajectories has significantly increased the complexity of the retirement transition. Consequently, retirement has become a less predictable and more individualized life-course milestone compared to earlier periods characterized by relatively fixed retirement ages.

In China, the proportion of aged population has been increasing in recent years. Data from successive national population censuses reveal that the share of individuals aged 60 and above has risen from 7.62% in 1982 to 18.70% in 2020, while the proportion of those aged 65 and above has grown from 4.91% to 13.5% over the same period [38]. Concurrently, the average life expectancy of the Chinese population has continued to rise, increasing from 69.27 to 80.88 years for women, and from 66.28 to 75.37 years for men, between 1981 and 2020 [39]. These trends indicate that an expanding number of people are entering old age, and their post-retirement years are becoming substantially longer. This extended period is particularly notable given China’s long-standing statutory retirement ages: 60 for men, 50 for female workers, and 55 for female cadres before 2025 (Starting from 2025, the legal retirement age in China has been adjusted to 63 for men, 55 for women, and 58 for female cadres)—ages which are considerably lower than the rising life expectancy. This growing gap between the legal retirement age and life expectancy creates a significant potential for human resource utilization in later life. Therefore, policy initiatives that encourage the reemployment of older adults have begun to emerge. A key example is the “Outline of the 14th Five-Year Plan (2021-2025) for National Economic and Social Development and Vision 2035 of the People’s Republic of China,” which explicitly states: “Taking into account factors such as rising average life expectancy, accelerating population aging, increasing years of education, and changes in the labor force structure, we will gradually delay the statutory retirement age in accordance with the principles of making incremental adjustments, implementing them flexibly, advancing in different categories, and planning as a whole, to promote the full utilization of human resources.” This framework provides a supportive policy environment for the return of older individuals to the labor market.

Within this context, what strategies do older adults adopt to navigate their extended late-life stage? National census data reveal that in 2000, the employed population aged 50 and above accounted for 0.98% of the total population, and those aged 60 and above accounted for 0.34%; by 2020, these figures had risen to 1.38% and 0.41%, respectively. Despite this increase, the labor force participation rate of older adults in China remains notably low compared to many developed nations. For example, Cheng and Li, [40] using the 2016 China Urban Labor Survey data, estimated the labor participation rate of the retired population in China at 4.2%, with the rate for those aged 65 and above at only 1.8%. Similarly, Feng et al. [41] found that retirement reduced the probability of labor market participation by 47% for urban male workers and 40% for urban female workers. Another study based on data from 2002–2009 China Urban Household Survey, suggested that only about 7% of urban workers re-entered the labor market after retirement [42]. However, emerging research points to growing complexity. Song et al. [6] have recently identified significant diversification in the retirement transition of urban older adults in China. Therefore, while extensive evidence documents delayed and diversified retirement in developed countries, scholarly understating of the evolving patterns and nature of retirement transitions in the Chinese context remain inconclusive and warrants further investigation.

### 2.3. Retirement and physical health

The relationship between retirement and subsequent health in later life remains controversial. Evidence from different countries indicates that retirement has a positive effect on health status. For instance, retirement has been linked to significant improvements in happiness and self-reported health in the UK [8], a positive impact on physical health in Norway [7], and overall better self-rated health in Italy [9]. Broader studies covering 11 European countries suggest that retirement has a protective effect, reducing the likelihood of reporting average to very poor health by 35% and improving health indices by nearly one standard deviation [43]. Similar findings from the United States provide robust evidence for enhanced self-reported health and life satisfaction following retirement [44]. Furthermore, meta-analyses have concluded that retirement significantly influences both overall and physical health [45], and that the transition from work to retirement is associated with better self-reported health, albeit alongside a decline in cognitive abilities [46]. Conversely, a substantial body of contrary evidence indicates that the average effect of retirement on health outcomes is often negligible. Specifically, studies highlight that negative health impacts are primarily associated with mandatory or involuntary retirement [47].

The relationship between retirement and health is further moderated by several key factors, including retirement age, retirement duration, the reason for retirement, and its type. Regarding retirement age, findings are mixed. Some studies indicate that men who retire earlier report better physical health [48], whereas retiring at the standard age may be associated with a higher mortality risk [49]. Other studies have found no impact of reaching the legal retirement age of 60 on physical functioning [23]. Delaying retirement appears to have a negative or negligible effect on self-reported health [46] yet offers a protective effect against cognitive decline, an effect most pronounced among individuals with the highest level of education [50]. However, the impact is not uniform across socioeconomic groups; raising the state pension age has been shown to have a significant negative impact on the physical health of British women from lower socioeconomic status [51]. Concerning the duration of retirement, evidence suggests that the potential health benefits of retirement may manifest slowly. Positive effects on health status often become apparent only after four or more years, supporting the view of health as a slowly evolving stock [44]. Conversely, other longitudinal studies tracking an average of six years post-retirement indicate that full retirement can lead to increased difficulties with mobility and daily activities, as well as a rise in disease conditions. These adverse effects, however, may be mitigated by lifestyle adaptations, such as maintaining physical activity after retirement [52].

The reason for retirement is a critical moderator of the retirement-health nexus, often closely tied to pre-retirement health status. Scholars have found that leaving the labor force for non-health reasons shows no significant association with subsequent health changes, but retirement driven by poor health is consistently linked to subsequent health deterioration [53]. Research based in Scotland has also found that the reason for retirement is a more significant predictor of post-retirement health than age itself, with health-related retirements associated with poorer physical and mental health outcomes [54]. Additionally, the planning process preceding retirement also matters, as it can indirectly or directly affects physical health and life satisfaction through its effect on retirement confidence [55]. Similarly, the type or pathway of retirement plays a defining role. Compared to a direct transition from full-time work to full retirement, pathways involving phased partial retirement are associated with poorer subsequent health [53]. Longitudinal studies show minimal change in physical functioning for those undergoing statutory or part-time retirement, whereas a significant decline is observed among disabled retirees [56]. Other studies have found that both statutory and early voluntary retirement are associated with improved health compared to continued employment, although this benefit may diminish over time [57]. Furthermore, the involuntariness of the retirement transition appears paramount: involuntary retirement is directly associated with worse subsequent self-rated health, while voluntary retirement is indirectly associated with better self-rated health, mediated by a greater sense of financial control [58]. Based on above, this study focuses on analyzing the diverse retirement transitions among middle-aged and older adults in China and their subsequent impact on health.

## 3. Analytical strategy

### 3.1. Data

The data for this study is sourced from the China Health and Retirement Longitudinal Study (CHARLS), a nationally representative longitudinal survey project administered by the National School of Development at Peking University. Targeting China’s middle-aged and older adults, CHARLS employs a multi-stage, stratified probability sampling design covering 28 provincial-level regions. Since the baseline survey in 2011, four follow-up surveys have been conducted in 2013, 2015, 2018, and 2020. With an initial response rate of 80.5%, this rich dataset is widely used by scholars to study aging and population development in China.

In 2014, CHARLS conducted a retrospective survey on the life course of Chinese older people, collecting life history information on respondents’ education, fertility, marriage, employment, and migration. 292 deceased respondents withdrew from the 2014 survey. This study primarily draws on the detailed work histories and retirement experiences recorded in this wave. In the process of handling missing data, this study deleted cases with incorrect information and missing values.

Since the transition to retirement mainly involves senior respondents who have retired or are about to face retirement, this study selected 12,580 older adults born between 1921 and 1960. This period was divided into six five-year birth cohorts (see Table 1) to facilitate a life-course perspective on retirement transitions. It should be noted that the two birth cohorts of 1951–1955 and 1956–1960 were not all aged 65 or above during the 2014 survey and did not cover all age points from 45 to 65 for these two cohorts. Nonetheless, they were included in the analysis to capture preliminary patterns in their early retirement transitions.

**Table 1:** Distribution of selected birth cohorts (N = 12580).

Cohorts	Age at the time of the survey in 2014	Gender	
		Men	Women
1921-1925 cohort	89-93	25 (0.40%)	50 (0.79%)
1926-1930 cohort	84-88	109 (1.74%)	138 (2.18%)
1931-1935 cohort	79-83	312 (4.98%)	304 (4.81%)
1936-1940 cohort	74-78	604 (9.64%)	493 (7.81%)
1941-1945 cohort	69-73	855 (13.65%)	849 (13.44%)
1946-1950 cohort	64-68	1337 (21.34%)	1251 (19.81%)
1951-1955 cohort	59-63	1660 (26.50%)	1835 (29.05%)
1956-1960 cohort	54-58	1362 (21.74%)	1396 (22.10%)
Total	54-93	6264 (100%)	6316 (100%)

Source: 2014 Life history survey data of China Health and Retirement Longitudinal Study.

### 3.2. Variables

#### 3.2.1. Dependent variable.

The dependent variable in this study is the physical health of middle-aged and older people, measured by four types of indicators: subjective self-rated health and Activities of Daily Living (ADL), Instrumental Activity of Daily Living (IADL), and Index of basic physical activities scale (NAGI). Subjective self-rated health is measured by the question “How do you feel about your current health status?” with response options including “Very good,” “Good,” “Average,” “Poor,” and “Very poor.” The ADL scale includes six items: eating, dressing, getting in or out of bed, bathing or showering, using the toilet, and controlling urination and defecation. The IADL scale includes five items: doing housework, cooking, shopping, managing money, and taking medication. The NAGI, proposed by Nagi [59], includes six items: the ability to stand up after sitting for a long time, climb stairs continuously, bend or squat, stretch arms, lift heavy objects, and pick up coins from a table. For the ADL, IADL, and NAGI scales, responses are coded as follows: “No, I don’t have any difficulty” = 1; “I have difficulty but can still do it” = 2; “Yes, I have difficulty and need help” = 3; and “I cannot do it” = 4. The total score for each scale (ADL, IADL, and NAGI) is calculated by summing the scores of its corresponding items. Higher scores indicate a higher degree of disability, that is, weaker daily living abilities.

#### 3.2.2. Independent variable and moderating variable.

The independent variable in our study is the transition to retirement group, derived from sequence analysis. The moderating variable in this study is contact with children, measured by two questions in the questionnaire: “How often do you see your children when you are not living with them?” and “How often do you communicate with your children through phone calls, text messages, WeChat, letters, or emails when you are not living with them?” Response options include: “Almost every day (=1),” “2-3 times a week (=2),” “Once a week (=3),” “Every two weeks (=4),” “Once a month (=5),” “Once every three months (=6),” “Once every six months (=7),” “Once a year (=8),” and “Almost never (=9).” Contact with children is obtained by taking the mean score of responses to the two questions above and is treated as a continuous variable. The maximum value of this variable is 9, and the minimum value is 1.

#### 3.2.3. Control variables.

Previous studies have found that there are significant individual differences in the physical health of older adults, which vary by gender, hukou (China’s household registration system), and education level [41,60,61]. Therefore, this study includes birth cohort, ethnicity, hukou, marital status, and education level as control variables in the analysis. Birth cohorts are categorized as 1921–1925 cohort (=1), 1926–1930 cohort (=2), 1931–1935 cohort (=3), 1936–1940 cohort (=4), 1941–1945 cohort (=5), 1946–1950 cohort (=6), 1951–1955 cohort (=7), and 1956–1960 cohort (=8). Ethnicity includes two categories: ethnic minorities (=1) and Han Chinese (=2). Hukou also includes two categories, with 1 representing rural hukou and 2 representing urban hukou. Marital status is divided into two categories: without a spouse (=1) and with a spouse (=2). Education level includes four categories: primary school or below (=1), junior high school (=2), high school (=3), and university or above (=4). Additionally, previous studies have shown that there is a link between pre-retirement health problems and subsequent poor health status [53]. Therefore, this study also includes the self-rated health variable at the time of retirement in the analysis (for respondents who are about to retire but have not yet retired, the subjective self-rated health at the time of the survey is used to fill in), to control for potential endogeneity issues.

### 3.3. Analytical methods

#### 3.3.1. Sequence analysis.

The sequence analysis method is used to describe the timing, state distribution, and the transition to retirement of middle-aged and older adults. Firstly, to analyze the retirement transition of respondents, it is necessary to define the age range for this transition. This study, considering the actual situation in China, determines the age range for retirement transition to be 45–65 years.

Secondly, the state space for retirement transition is defined. According to the state definitions in existing studies, although the state definitions vary, most focus on changes in work status and income. Therefore, this study also defines the state space for retirement transition from this perspective. Specifically, this study defines the following states: “Government & Public Institution”, “Non-agricultural Employment”, “Non-agricultural Self-employment”, “Agricultural employment”, “Own Agricultural Production”, “Army”, “Unpaid Work”, “Education/Training”, “Take Care of Family”, “Disability/Illness”, “Unemployed”, “Never worked”, “Retirement”, totaling 13 states.

Thirdly, retirement transition for different birth cohorts are formed. This study utilizes information from the work history survey module in the CHARLS 2014 retrospective survey data and the 13 states defined above to extract the employment status of Chinese middle-aged and older adults born between 1921 and 1960 at various ages from 45 to 65, forming sequences of retirement transition for different individuals with a half-year as the time unit.

Fourthly, the groups of retirement transition are identified through optimal matching and cluster analysis. Based on the generated retirement transition sequences, the distances between the retirement transition state sequences of respondents are calculated using the optimal matching method. Optimal matching is based on three procedures: insertion, deletion, and substitution, with a certain cost for sequence matching, thereby generating a distance matrix for different individual sequences. For each of the above operations, a specific cost must be set. This study follows the requirement that cost settings need to maintain non-triangular inequality, adopt the transition rate between states as the cost of substitution, and set the costs of insertion and deletion to a fixed value of 1.

Finally, we use the Ward algorithm for hierarchical cluster analysis to achieve data dimensionality reduction, thereby organizing retirement transition sequences into groups that maximize the similarity within groups and minimize it between groups. The validity of the clustering is tested through a series of indicators, indicating the stability of the clustering level and the maximum occupancy of case information.

#### 3.3.2. Multiple regression analysis.

This study uses the physical health of middle-aged and older adults as the dependent variable and adopts ordinal logistic regression and multiple linear regression methods to estimate the association between retirement transition and physical health. To examine the moderating role of contact with children on the relationship between retirement transition and the physical health of middle-aged and older adults, this study establishes an interaction term between retirement transition and contact with children, and calculates the marginal effect of the interaction term between retirement transition groups and contact with children on the dependent variable.

## 4. Results

### 4.1. Groups of transition to retirement

**Fig 1:**
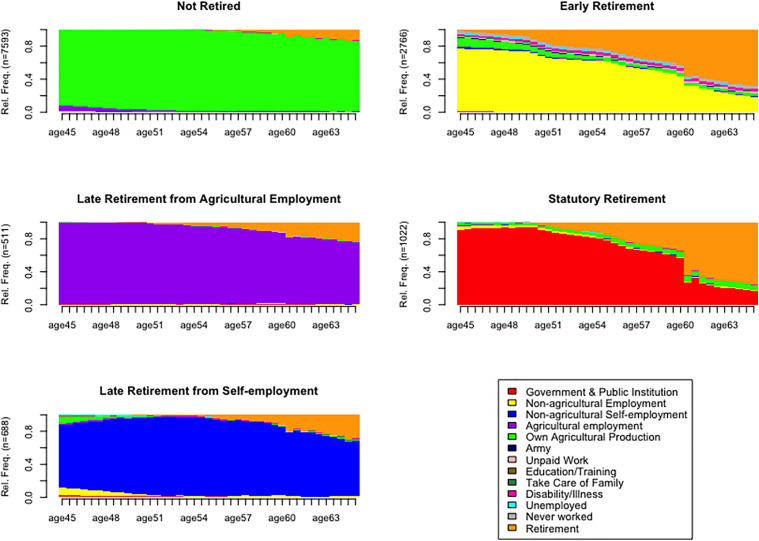
Groups of transition to retirement. A series of test indicators support a decision scheme that classifies the retirement transition into five clusters. For example, PBC is 0.88, the highest among all clustering schemes; HG and HGSD were both 0.96, also the highest among all clustering schemes. ASW and ASWw are both 0.68, which is also the highest in all clustering schemes. CH is 4388.24, CHsq is 10528.29, and the index value is also relatively high. Based on the above results, this study adopts the scheme of five-groups clustering scheme. Source: 2014 Life History Survey data of China Health and Retirement Longitudinal Study.

This study identified five distinct groups of retirement transition patterns. The state distribution for each group is presented in Fig 1. The first group is predominantly characterized by the state of “Own Agricultural Production”. Nearly all members have engaged in long-term farming, and a significant portion had not yet retired by the end of the observation period. Therefore, this group is named “Not Retired”. The second group is primarily defined by the states of “Non-agricultural Employment” and “Retirement”, indicating that most members of this group who transitioned from non-agricultural employment to retirement relatively early in their life course. Based on this characteristic, this group is named “Early Retirement”. The dominant state in the third group is “Agricultural Employment”. Members of this group followed a trajectory of prolonged engagement in agricultural work, extending into the later stages of their life course before retirement. We called it “Late Retirement from Agricultural Employment”. The fourth group features a high proportion of the states “Government & Public Institution” and “Retirement”. Members of this group have transitioned from employment in government or public institutions to retirement at the statutory age. Therefore, it is named “Statutory Retirement”. The fifth group is primarily characterized by the state of “Non-agricultural Self-employment”. It consists of self-employed individuals who gradually withdrew from the labor market after a long-term period of self-employment. We named it “Late Retirement from Self-employment”.

The “Not Retired” group comprises the largest proportion of the sample at 60.36%. This group is mainly composed of rural residents engaged in long-term agricultural production for a living, with retirement typically occurring very late in the life course. For example, at age 65, 85.80% of its members are still in the state of “Own Agricultural Production”, while only 12.68% have entered “retirement”. The group of “Early Retirement” represents the second-largest group at 21.99%. This group mainly includes non-agricultural employee, such as enterprise workers, who exit the labor market relatively early. By age 65, 67.59% of its members are already in “Retirement” state. The “Late Retirement from Agricultural Employment” group exhibits a delayed exit pattern similar to the first group. For instance, at age 65, only 22.71% of its members are retired, whereas 75.08% remain in “Agricultural Employment”. The “Statutory Retirement” group consists of “institutional” staff from government agencies and public institutions who generally retire at the statutory age. For example, with 75.76% of its members in the state of “Retirement” by age 65. The majority of members in the group of “Late Retirement from Self-employment” are mainly engaged in flexible self-employment work, with relatively free work forms and time, so they may retire at a later age. At age 65, only 28.51% are retired, while 66.97% continue in the state of “Non-agricultural Self-employment”.

Overall, the three groups of “Not Retired,” “Late Retirement from Agricultural Employment,” and “Late Retirement from Self-employment” exhibit delayed transition to retirement, typically exiting the labor market gradually in later life. This pattern is largely attributable to the flexible nature of their work (agricultural production or self-employment) and critically, the relatively weaker pension security support available to farmers and the self-employed. To secure their livelihood in old-age, individuals in these groups often need to prolong their working lives. Members of the two groups of “Statutory Retirement” and “Late Retirement from Self-employment” transition to retirement relatively earlier. These individuals are predominantly employed in formal “institutional” sectors, where strong pension benefits provide post-retirement security, and where relatively strict statutory retirement age requirements. Therefore, their retirement pathways tend to be more standardized and institutionally prescribed.

### 4.2. Health disparities among groups with different characteristics

Table 2 displays the health disparities among groups with different characteristics. Males have higher scores on ADL, IADL, NAGI, and self-rated health than females, indicating that males have a higher level of physical health than females. Older birth cohorts have significantly higher scores across all four health measures than younger cohorts, suggesting an overall decline in physical health levels in more recent generations. Ethnic minority respondents report higher scores than Han respondents on all health indicators. Respondents with agricultural *hukou* have higher ADL, IADL, NAGI, and self-rated health scores than those with non-agricultural *hukou*, indicating that respondents with non-agricultural *hukou* possess a higher level of physical health. Respondents with higher levels of education generally have lower scores across all four health measures compared to those with less education. Respondents without a spouse have higher health scores than those with a spouse, implying that married respondents have greater physical functional capabilities. Significant differences exist among retirement transition groups. The “Late Retirement from Agricultural Employment” group has the highest scores, followed by the group of “Not Retired,” while the “Late Retirement from Self-employment” group has the lowest scores across all health measures.

**Table 2:** Differences in physical health among different characteristic groups. Higher average scores for ADL, IADL, NAGI and self-rated health indicate poorer physical health.

Variables	ADL		IADL		NAGI		Self-rated health	
	Mean ± SD	P-value	Mean ± SD	P-value	Mean ± SD	P-value	Mean ± SD	P-value
Gender:								
Male	6.807 ± 2.205	*P* < 0.001	6.114 ± 2.867	*P* < 0.001	8.069 ± 3.236	*P* < 0.001	2.977 ± 0.966	*P* < 0.001
Female	6.970 ± 2.264		6.577 ± 3.126		9.397 ± 3.809		3.141 ± 0.942	
Age group:								
1921-1925	8.849 ± 4.001	*P* < 0.001	11.169 ± 5.554	*P* < 0.001	12.500 ± 5.395	*P* < 0.001	3.129 ± 0.796	*P* < 0.001
1926-1930	8.354 ± 3.684		9.500 ± 5.031		11.927 ± 4.588		3.117 ± 0.816	
1931-1935	7.775 ± 3.351		8.215 ± 4.493		10.875 ± 4.498		3.097 ± 0.927	
1936-1940	7.511 ± 3.057		7.353 ± 3.974		10.122 ± 4.342		3.153 ± 0.916	
1941-1945	7.121 ± 2.491		6.588 ± 3.165		9.418 ± 4.017		3.140 ± 0.924	
1946-1950	6.851 ± 2.129		6.230 ± 2.785		8.681 ± 3.523		3.133 ± 0.953	
1951-1955	6.597 ± 1.622		5.867 ± 2.244		8.097 ± 2.949		3.014 ± 0.974	
1956-1960	6.521 ± 1.622		5.683 ± 2.025		7.756 ± 2.625		2.944 ± 0.985	
Ethnicity:								
Ethnic Minority	7.227 ± 2.670	*P* < 0.001	6.741 ± 3.425	*P* < 0.001	9.097 ± 3.843	*P* = 0.0012	3.110 ± 0.994	*P* = 0.087
Han Nationality	6.861 ± 2.194		6.314 ± 2.969		8.706 ± 3.575		3.055 ± 0.954	
Hukou:								
Agricultural hukou	6.926 ± 2.285	*P* < 0.001	6.414 ± 3.067	*P* < 0.001	8.837 ± 3.670	*P* < 0.001	3.078 ± 0.963	*P* < 0.001
Non-agricultural hukou	6.581 ± 1.756		5.788 ± 2.398		7.893 ± 2.775		2.904 ± 0.898	
Educational level:								
Elementary and below	7.025 ± 2.366	*P* < 0.001	6.627 ± 3.236	*P* < 0.001	9.211 ± 3.826	*P* < 0.001	3.113 ± 0.966	*P* < 0.001
Middle school	6.598 ± 1.849		5.737 ± 2.320		7.728 ± 2.707		2.980 ± 0.924	
High school	6.537 ± 1.793		5.698 ± 2.269		7.614 ± 2.750		2.883 ± 0.944	
College or above	6.683 ± 2.418		5.606 ± 2.229		7.370 ± 2.831		2.837 ± 0.843	
Marital Status:								
Without a spouse	7.290 ± 2.648	*P* < 0.001	7.118 ± 3.690	*P* < 0.001	9.845 ± 4.101	*P* < 0.001	3.165 ± 0.930	*P* < 0.001
With a spouse	6.798 ± 2.122		6.172 ± 2.802		8.484 ± 3.424		3.035 ± 0.962	
Retirement transition groups:								
Not Retired	6.947 ± 2.208	*P* < 0.001	6.485 ± 3.032	*P* < 0.001	9.085 ± 3.728	*P* < 0.001	3.142 ± 0.963	*P* < 0.001
Early Retirement	6.790 ± 2.218		6.130 ± 2.955		8.171 ± 3.293		2.924 ± 0.945	
Late Retirement from Agricultural Employment	7.397 ± 3.081		7.412 ± 4.169		9.911 ± 4.238		3.123 ± 0.919	
Statutory Retirement	6.687 ± 2.221		5.825 ± 2.542		7.729 ± 2.955		2.904 ± 0.880	
Late Retirement from Self-employment	6.573 ± 1.751		5.676 ± 2.096		7.769 ± 2.696		2.867 ± 0.968	

Source: 2014 Life history survey data of China Health and Retirement Longitudinal Study.

### 4.3. The association between retirement transition and physical health

Table 3 presents the results of the association between retirement transition and respondents’ physical health, as well as the moderating role of contact with children, after controlling for s birth cohort, ethnicity, and hukou.

**Table 3:** Results of multiple regression analyzing the association between retirement and physical health in middle-aged and older adults.

	Model 1a: (ADL, main effect)	Model 1b: (ADL, interaction effect)	Model 2a: (IADL, main effect)	Model 2b: (IADL, interaction effect)	Model 3a: (NAGI, main effect)	Model 3b: (NAGI, interaction effect)	Model 4a: (Self-rated health, main effect)	Model 4b: (Self-rated health, interaction effect)
	B (SE)	B (SE)	B (SE)	B (SE)	B (SE)	B (SE)	OR (SE)	OR (SE)
**Independent variables and moderating variables:**								
Retirement transition groups: (Ref. = Not Retired)								
Early Retirement	0.036 (0.054)	−0.004 (0.159)	−0.016 (0.071)	−0.185 (0.210)	−0.345*** (0.083)	−0.533* (0.263)	0.759*** (0.034)	0.900 (0.133)
Late Retirement from Agricultural Employment	0.212* (0.100)	0.862*** (0.241)	0.407** (0.131)	1.151*** (0.317)	0.198 (0.154)	0.195 (0.397)	0.930 (0.077)	0.946 (0.203)
Statutory Retirement	−0.145† (0.082)	−0.476† (0.278)	−0.393*** (0.107)	−1.059** (0.366)	−0.791*** (0.126)	−1.271** (0.459)	0.733*** (0.049)	0.784 (0.197)
Late Retirement from Self-employment	−0.135 (0.088)	−0.463 (0.319)	−0.356** (0.116)	−0.532 (0.420)	−0.555*** (0.136)	−0.887† (0.526)	0.675*** (0.050)	1.031 (0.313)
Self-rated health at the time of retirement:	−0.157*** (0.043)	−0.119* (0.051)	−0.286* (0.056)	−0.237* (0.067)	−0.435*** (0.065)	−0.408*** (0.084)	0.636*** (0.023)	0.663*** (0.032)
Contact with children:		−0.038** (0.012)		−0.116*** (0.016)		−0.105*** (0.021)		0.968** (0.011)
Retirement transition groups x Contact with children: (Ref. = Not Retired x Contact with children)								
Early Retirement x Contact with children		0.007 (0.025)		0.022 (0.033)		0.035 (0.042)		0.965 (0.023)
Late Retirement from Agricultural Employment x Contact with children		−0.123** (0.045)		−0.148* (0.059)		0.002 (0.074)		1.008 (0.041)
Statutory Retirement x Contact with children		0.052 (0.042)		0.114* (0.055)		0.109 (0.069)		0.989 (0.038)
Late Retirement from Self-employment x Contact with children		0.071 (0.049)		0.077 (0.065)		0.087 (0.081)		0.946 (0.044)
Constant	9.166*** (0.264)	8.573*** (0.284)	11.433*** (0.345)	11.507*** (0.373)	12.425*** (0.405)	12.214*** (0.468)		
R2/ Pseudo R2	0.047	0.054	0.102	0.127	0.135	0.148	0.010	0.010
F/LR Value	34.74***	20.11***	78.93***	51.79***	109.08***	61.89***	384.08***	245.70***

Note: † p < 0.1; * p < 0.05; ** p < 0.01; *** p < 0.001.

Source: 2014 Life History Survey data of China Health and Retirement Longitudinal Study.

Regarding the control variables, as shown in Models 1–4 (see S1 Table), gender, ethnicity, *hukou*, education level, and marital status all demonstrate statistically significant associations with respondents’ physical health. Female respondents report lower levels of physical health than their male counterparts. Earlier birth cohorts generally exhibit lower physical health compared to later birth cohorts. Han respondents have higher physical health levels than respondents from ethnic minority groups. Respondents with urban *hukou* show better physical health than those with rural *hukou*. Respondents with a junior high school, high school, or college education and above have higher physical health scores than those with a primary school education or lower. Respondents with a spouse have higher physical health than those without a spouse. Respondents who self-reported being in good health at or before retirement have lower scores on the ADL, ADL2, NAGI, and self-rated health.

Regarding the retirement transition variable (Models 1a to 4a), significant health disparities are observed when compared to the “Not Retired” reference group. The “Early Retirement” group shows a lower NAGI score (*B* = −0.345) and a reduced probability of self-rated unhealthy status. The “Late Retirement from Agricultural Employment” group has higher ADL (*B* = 0.212) and IADL (*B* = 0.407) scores, indicating that a lower level of physical health compared to the reference group. Members of the “Statutory Retirement” group have significantly lower scores across ADL (*B* = 0.145), IADL (*B* = 0.393), and NAGI (*B* = 0.791) measures, alongside a lower probability of self-reporting poor health (OR= 0.733), collectively pointing to a higher level of physical health. The “Late Retirement from Self-employment” group exhibits lower IADL (*B* = −0.356) and NAGI (*B* = −0.555) scores, and a higher probability of self-reporting as unhealthy, indicating that members of the “Late Retirement from Self-employment” group have higher health than those of the “Not Retired” group.

Models 1b to 4b introduce the moderating variable of contact with children. The results show that contact with children is significant association with respondents’ physical health. For each unit increase in contact with children, respondents’ ADL, IADL, and NAGI scores decrease by 0.038, 0.116, and 0.105, respectively, and the probability of self-reporting as unhealthy also declines. In other words, respondents who have more frequent contact with their children have higher physical health.

The analysis of interaction effects reveals that contact with children moderates the relationship between retirement transition and health. Compared to the “Not Retired” group, for each additional unit of contact with children, the ADL and IADL scores of respondents in the “Late Retirement from Agricultural Employment” group decrease by 0.123 and 0.148, respectively, while the IADL score of respondents in the “Statutory Retirement” group increases by 0.114. This finding indicates that increased contact with children serves as a significant protective factor, moderating and enhancing the health outcomes specifically for individuals in the “Late Retirement from Agricultural Employment” group. In sum, members of the “Statutory Retirement” and “Late Retirement from Self-employment” groups exhibit higher health levels, whereas those in the “Late Retirement from Agricultural Employment” group demonstrate lower health levels. The moderating effect of children’s contact is most pronounced and beneficial for the latter group.

To further illustrate the moderating effect, S1 and S2 Figs present the marginal effects of the interaction between retirement transition groups and contact with children on physical health. The visualization confirms that the health benefit of increased contact with children is not uniform across retirement groups. Specifically, the increased contact with children has a greater effect on improving the health of respondents in the “Late Retirement from Agricultural Employment” group. Similarly, though less pronounced, improving effect is observed for the “Not Retired” and “Early Retirement” groups. In contrast, the health benefit associated with increased contact is not statistically evident for the “Statutory Retirement” group.

## 5. Conclusion and discussion

The transition from work to retirement constitutes a pivotal shift in later life [3]. Focuses on this process, this study utilizes retrospective data from the 2014 CHARLS to examine distinct patterns of retirement transition and their association with subsequent physical health. First, cluster analysis identified five groups of retirement transition: “Not Retired,” “Early Retirement,” “Late Retirement from Agricultural Employment,” “Statutory Retirement,” “Late Retirement from Self-employment”. Members of the “Statutory Retirement” and “Early Retirement” groups typically complete the retirement transition earlier in their life course. The often retire at the statutory age with robust pension support, following a more institutionalized retirement pathway. In contrast, members of the “Not Retired,” “Late Retirement from Agricultural Employment,” and “Late Retirement from Self-employment” groups transition out of the labor force later. Primarily engaged in agricultural production or self-employment—occupations characterized by flexible work arrangements but weaker pension security—they exemplify a more individualized or non-standardized retirement model. Second, multiple regression analysis indicate that retirement transition groups are significantly associated with physical health. Compared to the “Not Retired” group, both the “Statutory Retirement” and “Late Retirement from Self-employment” groups are associated with better health outcomes. Conversely, the “Late Retirement from Agricultural Employment” group is associated with poorer health. Finally, marginal effect analysis suggests that increased contact with children has a greater positive correlation with health for respondents in the “Late Retirement from Agricultural Employment” group and a modest benefit for those in the “Not Retired” and “Early Retirement” groups. However, no significant health improvement associated with increased contact is observed for the “Statutory Retirement” group.

This finding partially aligns with prior research documenting delayed and diversified retirement patterns in developed countries, where retirees often engage in part-time work [15,62]. However, the Chinese context reveals distinct pathways shaped by institutional structures. Retirees from government, institutional, or formal non-agricultural sectors, benefiting from pension security, typically follow an institutionalized path and largely withdraw from the labor market. However, individuals engaged in agricultural production or self-employment, lacking strong pension coverage, often follow an autonomous, prolonged retirement model, frequently working until later in life.

Scholars have also found that retirement is significantly associated with subsequent health [7,9,49]. This study, based on empirical data from China, supports this finding that the type of retirement has a significant impact on subsequent health status. However, this study further finds that members of the “Late Retirement from Agricultural Employment” group have the poorest health levels, and increased contact with children has the greatest improving effect on the health of this group of members. Therefore, for individuals engaged in agricultural employment and retiring later in their life course, strengthening ties with their children is crucial. This may be because middle-aged and older adults engaged in agricultural employment have low incomes, and visits from their children can provide material and psychological support to retired parents and offer potential care services, thereby promoting the physical health of retired parents.

The transition from work to retirement constitutes a critical juncture in the individual life course, shaped by the interplay between institutional structures and personal agency. Based on an empirical analysis within the Chinese context, this study reveals that individuals in the “Statutory Retirement” group follow an institutionalized and standardized life course. Their retirement timing, economic security, and social role transitions are largely predefined by clear institutional arrangements, facilitating a relatively smooth health transition. In contrast, groups such as “Late Retirement from Agricultural Employment” experience a de-standardized and individualized life course. The lack of institutional pension support compels them to extend their working lives, and their retirement decisions are predominantly constrained by economic necessity and familial support—highly individualized factors. These findings provide strong support for the “cumulative advantage/disadvantage” mechanism central to life course theory. Early-life institutional inequalities—such as those embedded in the urban-rural divide and occupational segmentation—are further compounded through the pivotal transition of retirement, culminating in pronounced health disparities in later life.

This study has several limitations. First, at the time of the survey, individuals from the 1951–1955 and 1956–1960 birth cohorts had not yet reached age65. Consequently, the data capture only a partial segment of their retirement transition, not the complete pathway from work to full retirement. Second, the retrospective nature of the life history data introduces the potential for recall bias, as respondents were required to report events from years past. Despite careful data cleaning, issues related to recall bias may still affect the validity of the study’s findings to some extent. Third, the exclusion of both attrition cases and missing data may affect the validity of the results. Attrition not only reduces the effective sample size but also introduce selection bias, potentially compromising the sample’s representativeness. Specifically, if individuals in poorer health are more likely to drop out, the analytical sample may overrepresent healthier populations, leading to an underestimation of the negative health consequences associated with retirement.

The findings of this study have important value for policy improvement. First, policymakers and service providers should enhance public awareness through education and training, helping future retirees understand the importance of retirement planning. This involves encouraging early lifestyle adjustments and the formulation of post-retirement plans to better prepare for this transition [55]. Second, policymakers could introduce more flexible retirement arrangements, such as phased retirement programs and services that support the continued employment or re-employment of middle-aged and older adults. Third, government agencies and social organizations should develop targeted interventions to address the specific needs of individuals undergoing retirement transition and urge adult children to maintain stronger contact and provide care for their retired parents.

## Supporting information

S1 Fig Marginal effect of contact with children on the relationship between retirement transition groups and ADL. (TIF)

S2 Fig Marginal effect of contact with children on the relationship between retirement transition groups and IADL. (TIF)

S1 Table Full regression results including all covariates. (DOCX)
