# Supplementary figures and images for: The transition to retirement and subsequent physical health among middle-aged and older adults in China: A life course perspective
Source: PLoS One. 2026 Apr 24;21(4):e0347550. doi: 10.1371/journal.pone.0347550 (PMC13108804; doi:10.1371/journal.pone.0347550)

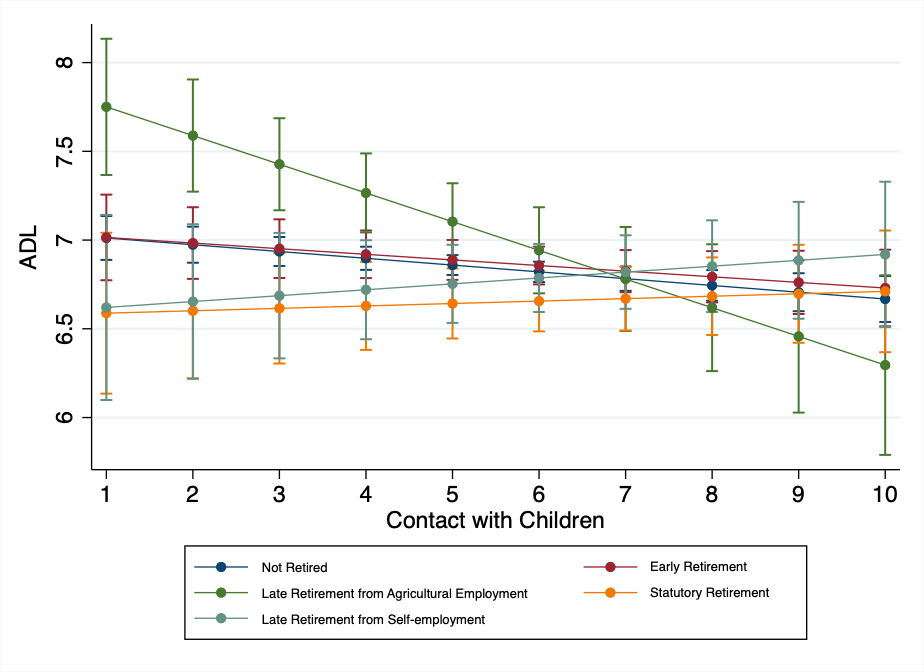

Supplement: S1 Fig — (TIF) [file pone.0347550.s001.tif]

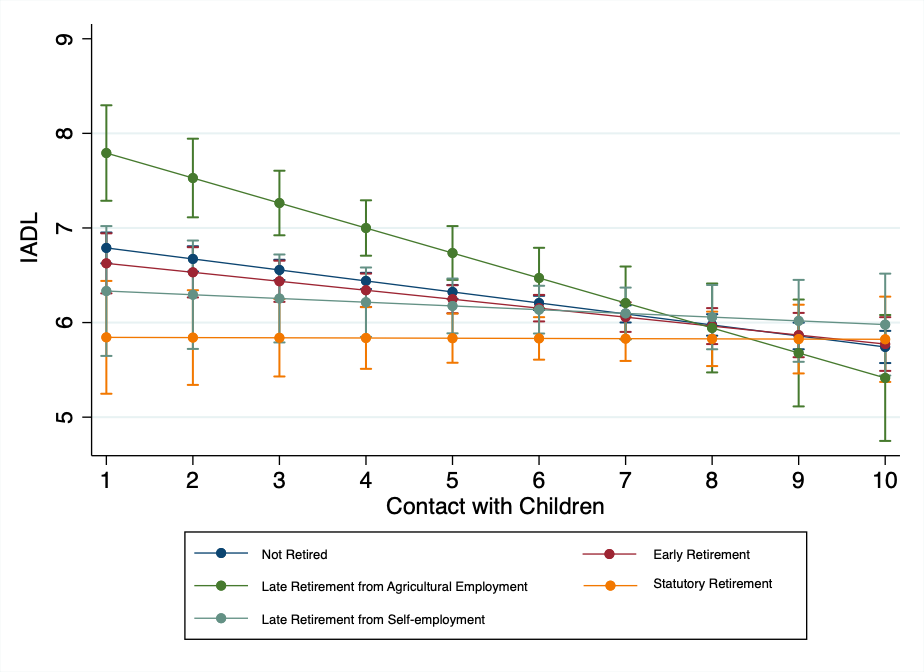

Supplement: S2 Fig — (TIF) [file pone.0347550.s002.tif]
