# Supplementary material for: The transition to retirement and subsequent physical health among middle-aged and older adults in China: A life course perspective
Source: PLoS One. 2026 Apr 24;21(4):e0347550. doi: 10.1371/journal.pone.0347550 (PMC13108804; doi:10.1371/journal.pone.0347550)
Supplement: S1 Table — (DOCX) [file pone.0347550.s003.docx]

**Table S1. Full regression results including all covariates**

|  | Model 1a: (ADL, main effect) | Model 1b: (ADL, interaction effect) | Model 2a: (IADL, main effect) | Model 2b: (IADL, interaction effect) | Model 3a: (NAGI, main effect) | Model 3b: (NAGI, interaction effect) | Model 4a:  (Self-rated health, main effect) | Model 4b:  (Self-rated health, interaction effect) |
| --- | --- | --- | --- | --- | --- | --- | --- | --- |
|  | B  (SE) | B  (SE) | B  (SE) | B  (SE) | B  (SE) | B  (SE) | OR  (SE) | OR  (SE) |
| Gender:（Ref.= Male） | 0.110**  (0.041) | 0.163***  (0.047) | 0.331***  (0.053) | 0.422***  (0.061) | 1.101***  (0.063) | 1.234***  (0.077) | 1.241***  (0.042) | 1.252***  (0.055) |
| Birth cohort: (Ref.=1921-1925 cohort) |  |  |  |  |  |  |  |  |
| 1926-1930 cohort | -0.461  (0.288) | 0.103  (0.300) | -1.583***  (0.376) | -1.531***  (0.395) | -0.405  (0.441) | -0.131  (0.459) | 1.014  (0.227) | 0.890  (0.232) |
| 1931-1935 cohort | -0.997***  (0.268) | -0.594*  (0.281) | -2.795***  (0.350) | -2.825***  (0.370) | -1.336***  (0.410) | -1.015*  (0.464) | 0.961  (0.201) | 0.876  (0.214) |
| 1936-1940 cohort | -1.242***  (0.262) | -0.760**  (0.276) | -3.608***  (0.342) | -3.557***  (0.363) | -1.972***  (0.401) | -1.688***  (0.455) | 0.989  (0.202) | 0.925  (0.221) |
| 1941-1945 cohort | -1.615***  (0.259) | -0.963***  (0.274) | -4.353***  (0.339) | -3.985***  (0.361) | -2.681***  (0.398) | -2.149***  (0.452) | 0.886  (0.179) | 0.832  (0.198) |
| 1946-1950 cohort | -1.877***  (0.258) | -1.180***  (0.273) | -4.698***  (0.337) | -4.224***  (0.360) | -3.390***  (0.395) | -2.772***  (0.451) | 0.869  (0.174) | 0.895  (0.212) |
| 1951-1955 cohort | -2.127***  (0.258) | -1.333***  (0.274) | -5.056***  (0.337) | -4.529***  (0.360) | -3.973***  (0.395) | -3.178***  (0.452) | 0.687†  (0.140) | 0.761  (0.181) |
| 1956-1960 cohort | -2.162***  (0.259) | -1.388***  (0.276) | -5.154***  (0.338) | -4.588***  (0.363) | -4.152***  (0.397) | -3.386***  (0.455) | 0.637*  (0.128) | 0.696  (0.167) |
| Ethnicity: (Ref.= Ethnic Minority) | -0.397***  (0.073) | -0.334***  (0.080) | -0.480***  (0.095) | -0.509***  (0.106) | -0.435***  (0.112) | -0.344**  (0.132) | 0.883*  (0.055) | 0.885  (0.067) |
| Hukou: (Ref.= Agricultural hukou) | -0.259***  (0.071) | -0.192*  (0.090) | -0.387***  (0.093) | -0.299*  (0.119) | -0.353***  (0.109) | -0.355*  (0.149) | 0.922  (0.054) | 0.884  (0.074) |
| Educational level: (Ref.= Elementary and below) |  |  |  |  |  |  |  |  |
| Middle school | -0.148**  (0.055) | -0.130*  (0.062) | -0.327***  (0.072) | -0.278***  (0.082) | -0.534***  (0.085) | -0.517***  (0.102) | 0.944  (0.043) | 0.906†  (0.053) |
| High school | -0.154*  (0.073) | -0.169*  (0.083) | -0.252**  (0.095) | -0.152  (0.110) | -0.439***  (0.112) | -0.445***  (0.138) | 0.847**  (0.052) | 0.834*  (0.065) |
| College or above | -0.086  (0.145) | -0.268  (0.179) | -0.447*  (0.189) | -0.474*  (0.236) | -0.753***  (0.222) | -1.097***  (0.295) | 0.817†  (0.096) | 0.843  (0.136) |
| Marital Status: (Ref.=without a spouse) | -0.068  (0.054) | -0.157**  (0.058) | -0.088  (0.070) | -0.184*  (0.076) | -0.198*  (0.082) | -0.282**  (0.095) | 0.847***  (0.038) | 0.937*  (0.051) |
| Retirement transition groups: (Ref.= Not Retired) |  |  |  |  |  |  |  |  |
| Early Retirement | 0.036  (0.054) | -0.004  (0.159) | -0.016  (0.071) | -0.185  (0.210) | -0.345***  (0.083) | -0.533*  (0.263) | 0.759***  (0.034) | 0.900  (0.133) |
| Late Retirement from Agricultural Employment | 0.212*  (0.100) | 0.862***  (0.241) | 0.407**  (0.131) | 1.151***  (0.317) | 0.198  (0.154) | 0.195  (0.397) | 0.930  (0.077) | 0.946  (0.203) |
| Statutory Retirement | -0.145†  (0.082) | -0.476†  (0.278) | -0.393***  (0.107) | -1.059**  (0.366) | -0.791***  (0.126) | -1.271**  (0.459) | 0.733***  (0.049) | 0.784  (0.197) |
| Late Retirement from Self-employment | -0.135  (0.088) | -0.463  (0.319) | -0.356**  (0.116) | -0.532  (0.420) | -0.555***  (0.136) | -0.887†  (0.526) | 0.675***  (0.050) | 1.031  (0.313) |
| Self-rated health at the time of retirement: | -0.157***  (0.043) | -0.119*  (0.051) | -0.286*  (0.056) | -0.237*  (0.067) | -0.435***  (0.065) | -0.408***  (0.084) | 0.636***  (0.023) | 0.663***  (0.032) |
| Contact with children: |  | -0.038**  (0.012) |  | -0.116***  (0.016) |  | -0.105***  (0.021) |  | 0.968**  (0.011) |
| Retirement transition groups x Contact with children: (Ref.= Not Retired x Contact with children) |  |  |  |  |  |  |  |  |
| Early Retirement x Contact with children |  | 0.007  (0.025) |  | 0.022  (0.033) |  | 0.035  (0.042) |  | 0.965  (0.023) |
| Late Retirement from Agricultural Employment x Contact with children |  | -0.123**  (0.045) |  | -0.148*  (0.059) |  | 0.002  (0.074) |  | 1.008  (0.041) |
| Statutory Retirement x Contact with children |  | 0.052  (0.042) |  | 0.114*  (0.055) |  | 0.109  (0.069) |  | 0.989  (0.038) |
| Late Retirement from Self-employment x Contact with children |  | 0.071  (0.049) |  | 0.077  (0.065) |  | 0.087  (0.081) |  | 0.946  (0.044) |
| Constant | 9.166***  (0.264) | 8.573***  (0.284) | 11.433***  (0.345) | 11.507***  (0.373) | 12.425***  (0.405) | 12.214***  (0.468) |  |  |
| R2/ Pseudo R2 | 0.047 | 0.054 | 0.102 | 0.127 | 0.135 | 0.148 | 0.010 | 0.010 |
| F/LR Value | 34.74*** | 20.11*** | 78.93*** | 51.79*** | 109.08*** | 61.89*** | 384.08*** | 245.70*** |

Note: † p<0.1; * p<0.05; ** p<0.01; *** p<0.001.

Source: 2014 Life History Survey data of China Health and Retirement Longitudinal Study.
